# Community-based serum chloride abnormalities predict mortality risk

**DOI:** 10.1371/journal.pone.0279837

**Published:** 2023-02-21

**Authors:** Tali Shafat, Victor Novack, Leonid Barski, Yosef S. Haviv

**Affiliations:** 1 Clinical Research Center, Soroka University Medical Center and the Faculty of Health Sciences, Ben-Gurion University of the Negev, Beer Sheba, Israel; 2 Department of Internal Medicine F, Soroka University Medical Center and the Faculty of Health Sciences, Ben-Gurion University of the Negev, Beer Sheba, Israel; 3 Nephrology Department, Soroka University Medical Center and the Faculty of Health Sciences, Ben-Gurion University of the Negev, Beer Sheba, Israel; International University of Health and Welfare, School of Medicine, JAPAN

## Abstract

**Introduction:**

This population-based study aimed to investigate the prognostic value of ambulatory serum chloride abnormalities, often ignored by physicians.

**Methods:**

The study population included all non-hospitalized adult patients, insured by "Clalit" Health Services in Israel’s southern district, who underwent at least 3 serum chloride tests in community-based clinics during 2005–2016. For each patient, each period with low (≤97 mmol/l), high (≥107 mmol/l) or normal chloride levels were recorded. A Cox proportional hazards model was used to estimate the mortality risk of hypochloremia and hyperchloremia periods.

**Results:**

664,253 serum chloride tests from 105,655 subjects were analyzed. During a median follow up of 10.8 years, 11,694 patients died. Hypochloremia (≤ 97 mmol/l) was independently associated with elevated all-cause mortality risk after adjusting for age, co-morbidities, hyponatremia and eGFR (HR 2.41, 95%CI 2.16–2.69, p<0.001). Crude hyperchloremia (≥107 mmol/L) was not associated with all-cause mortality (HR 1.03, 95%CI 0.98–1.09 p = 0.231); as opposed to hyperchloremia ≥108 mmol/l (HR 1.14, 95%CI 1.06–1.21 p<0.001). Secondary analysis revealed a dose-dependent elevated mortality risk for chloride levels of 105 mmol/l and below, well within the "normal" range.

**Conclusion:**

In the outpatient setting, hypochloremia is independently associated with an increased mortality risk. This risk is dose-dependent where the lower the chloride level, the higher is the risk.

## Introduction

Chloride is the major extracellular anion playing a key role in acid-base balance and body fluid homeostasis, distinctly from sodium. Nevertheless, serum chloride levels are often ignored despite their availability in routine chemistry blood tests. Recently, several studies among chronic heart failure patients indicated that chronic hypochloremia can serve as a prognostic marker for all-cause mortality, even better than hyponatremia [1–6]. Similar associations between hypochloremia and all-cause mortality and cardiovascular events were also found among chronic kidney disease (CKD) patients and hypertensive patients [7–9]. However, not all studies reported a methodology for the adjustment for concomitant hyponatremia, a known dominant risk factor for mortality, and except for 2 studies [1, 10], all studies examined specific patient populations rather than the general population [2–9]. The first study evaluating the prognostic value of serum chloride levels in the general outpatient population originated as an unexpected finding of a nutrition study conducted between 1979 to 1985 [1]. The second study from Mayo clinic examined a large cohort and found a U-shaped association between serum chloride levels before discharge and 1- year outcome where optimal serum chloride levels were 100–108 mmol/l [10].

Different values for the normal ranges for serum chloride have been previously reported: 100–108 mmol/L, 97–110 mmol/L or 98–106 [11, 12, 19]. It had been suggested that chloride <100 mmol/L or <102.8 is associated with higher mortality [3, 7] and in another large prospective study the lowest and highest chloride level quartiles (<104 mmol/L and >108 mmol/L respectively) were both associated with higher all-cause mortality risk and CV events [9].

In addition, while hyperchloremia confers a mortality risk in acutely ill ICU patient [13–16], it is currently unknown whether chronic hyperchloremia can serve as independent prognostic marker. A recent study among CKD patients’ cohorts showed that a serum chloride level of 106–108 is associated with the lowest mortality risk [8].

Finally, because in the primary care settings blood gases measurement is commonly not available, we hypothesized that community-based serum chloride test can serve as a simple and accessible proxy for blood pH [17, 18]. Taken together, the drivers to study the prognostic value of serum chloride were i. to generate a large updated population-based database from the general population to evaluate the risk associated with serum chloride abnormalities. ii. to relate the acid base status with the serum chloride level. This population-based study aimed to investigate these 2 parameters and is the first hospital-excluding study designed to characterize the significance of serum chloride levels in the general community population.

## Materials and methods

### Study population

This population-based study included all adult patients, insured by "Clalit" health plan, Israel southern district, who underwent at least three serum chloride tests during 2002–2016. Clalit is an extensive health plan, insuring 4,972,000 people in Israel, where its south district has 564,000 members. As the focus of this study was the prognostic value of ambulatory serum chloride levels, we excluded tests taken during hospitalization or in emergency room, as well as tests taken one week before or after hospitalization. Other exclusion criteria included patients with chronic diseases that potentially alter serum chloride levels, including ESRD patients (according to ICD-9 diagnoses, dialysis or kidney transplant), multiple myeloma and TPN-dependent patients, patients who underwent Ileal conduit urinary diversion and patients with chronic diarrhea or ileostomy, and age<18 yr.

Clinical and laboratory data were retrieved from Clalit computerized databases. Parameters collected comprised demographics, chronic comorbidities (according to diagnoses recorded by ICD-9), laboratory tests and medications taken for three months as evident by pharmacy purchase. The study was approved by the Institutional Review Board Committee of Soroka University Medical Center, waiving the need for informed consent due to the retrospective nature of the study. All methods were carried out in accordance with the institutional guidelines and regulations.

### Serum chloride measurement

The normal range for serum chloride level in our regional main laboratory is 98–106 mmol/l, as suggested by the *Mosby’s Diagnostic and Laboratory Test Reference*. 14th ed. St. Louis, MO: Elsevier; 2019. 233 [19]. Of note, some previous reports adopted this normal range [20], while other studies selected 96 mmol/l [6] or 95 [21, 22] as the lowest normal level. Of special note is the following methodology we employed to evaluate the significance of hypochloremia in the community. While hypochloremia (or hyponatremia) in hospitalized patients is a singular event thereby allowing direct patient-centered comparison, for the community-based analysis we employed chloride test-centered comparison over a number of years because serum chloride levels may vary and shift between normal and abnormal levels over the years. Thus, for each patient, all chloride tests taken during follow up were analyzed, and categorized as low (≤97 mmol/L)), high (≥107 mmol/L) or normal (98–106 mmol/L). Consequently, our study design did not include fixed distinct study groups (hypochloremia /euchloremia /hyperchloremia) because each patient could be included in different chloride groups at different time points. To balance these differences we used multivariate analysis, and subgroup analysis according to comorbidities and drug therapy as described in the in S1 File. Patient follow up duration was divided into chloride test intervals. Every chloride test value was considered to be active until the next test was taken, assuming that abnormal test levels were taken more frequently than normal range results. For each chloride test, we collected and adjusted the most timely-related serum sodium, serum creatinine, serum albumin and blood gas tests. Furthermore, we combined active medical diagnoses and chronic medication use with the corresponding timely serum chloride level that was measured at that time period. Coefficient of variation of chloride levels was calculated by the standard deviation divided by mean of all chloride tests for any given subject.

### Outcome definitions

Outcome was defined as all-cause mortality. Secondary analysis evaluated the risk associated with consecutive serum chloride levels within the normal range [98–106 mmol/L]. Association between acid-base abnormalities and serum chloride level comprised the definition of metabolic acidosis as both pH<7.38 and HCO3<23, and metabolic alkalosis as both pH>7.42 and HCO3>30, proportions in every chloride group, and correlation between serum bicarbonate and chloride levels.

### Statistical analysis

The statistics for continuous variables included mean, standard deviation, minimum, maximum, and sample. Categorical variables were described with numbers and percentages. We used generalized estimating equation (GEE) logistic regression model (unstructured matrix) to compare proportions of baseline characteristics between serum chloride groups (hypochloremia, hyperchloremia and euchloremia) to account for the clustering in the same patient. We utilized a patient-test as a unit of analysis; i.e. each patient could be presented by a number of test results. This approach allowed the utilization of a number of observations rather then only one elected observation or the mean for a given patient. A Cox proportional hazards model with patient level clustering and robust estimates was used to evaluate the mortality risk of hyperchloremia and hypochloremia periods adjusted to age, sex, co-morbidities, serum sodium level, albumin level and eGFR (MDRD- **eGFR** = 175 x (SCr)-1.154 x (age)-0.203 x 0.742 [if female] x 1.212 [if Black]), diuretics and RAS inhibitors therapy.

Because hypochloremia and hyponatremia emerged as the major risk factors, and because water balance affects serum osmolarity, thereby affecting both serum sodium and chloride levels, we further stratified hypochloremia and hyponatremia results into 4 groups, as previously suggested [21, 23]. Hyponatremic hypochloremia (unknwon chloride status), hypochloremia/eunatremia (chloride-depletion hypochloremia), hyponatremia/euchloremia (relative hyperchloremia), and neither hypochloremia nor hyponatremia (normal reference). Hypochloremia and hypernatremia are practically non-existent together (as evident in the open database accompanying this publication). Graphic presentation of serum venous blood HCO_3_ and chloride correlation used the LOWESS (Locally Weighted Scatterplot Smoothing) method.

All statistical tests were considered significant at α = 0.05 (2-sided) except for those specified otherwise. All p-values reported were rounded to three decimal places. Data summaries were performed using SPSS 25.0 statistical software (IBM Corp Armonk, NY, USA) and STATA (version 12.0).

## Results

### Population

**Fig 1:**
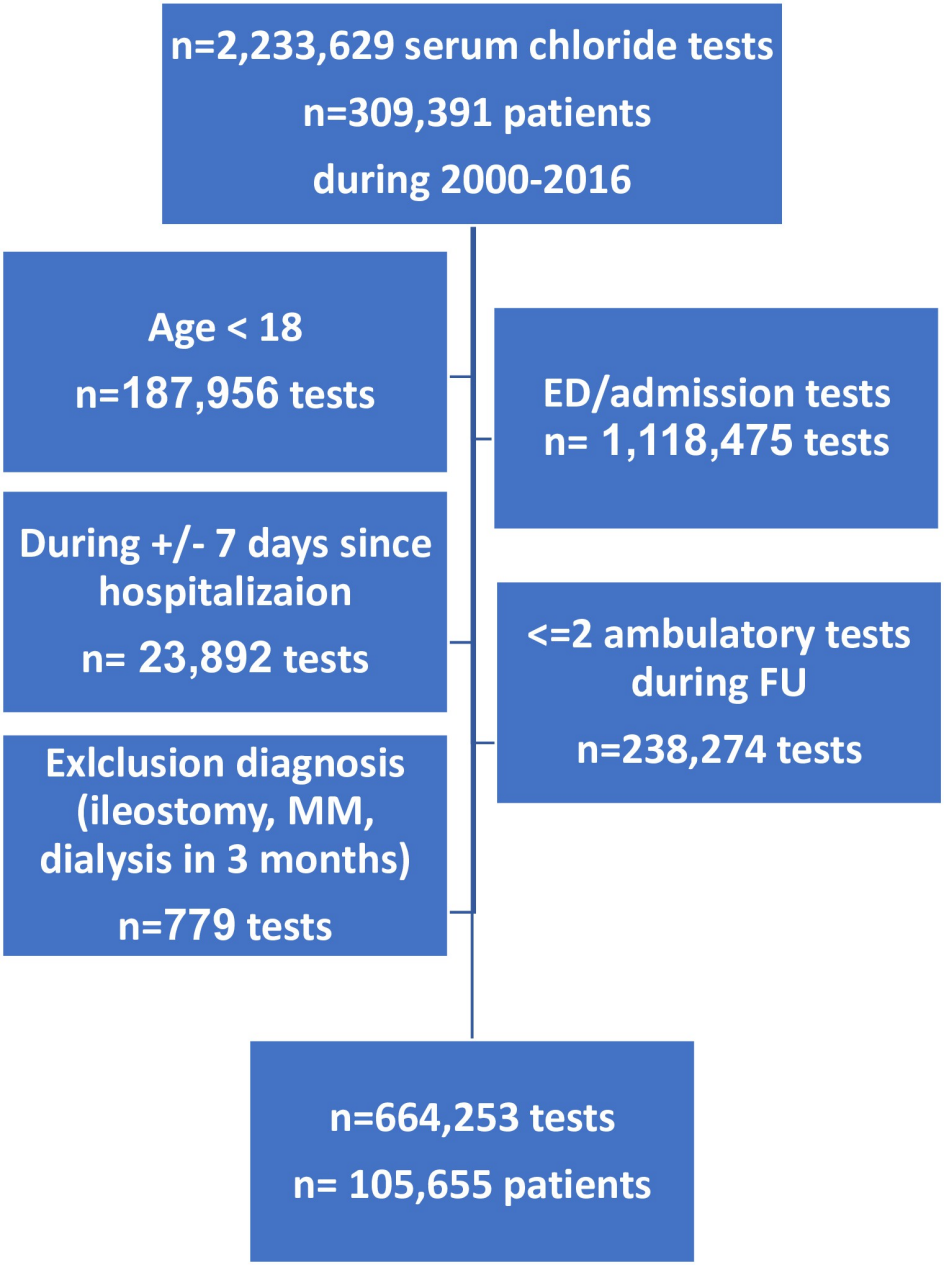


We collected the results all of 2,233,629 serum chloride tests from 309,391 patients performed during the years 2002–2016. After exclusion (Fig 1), 664,253 tests of 105,655 patients were included in the analysis.

Table 1 depicts demographic and comorbidity characteristics of the study population. Patients with laboratory episodes of hypochloremia were older (mean age 65.5 vs. 59.4 p = 0.005), more often had hypoalbuminemia (14.8% vs. 6.0% p = 0.001), had higher rates of cardiovascular disease, CHF and CKD (12.9% vs.7.3% p = 0.002, 8.1% vs. 2.8% p<0.001 and 8.2% vs. 4.5% p<0.001, respectively). Of note, CKD was associated with both hypo-and hyperchloremia indicating a U-shaped curve. Hyperchloremic patients were more often of female gender (61.6% vs. 55.9 p<0.001) and had higher rates of CKD and nephrolithiasis (8.6% vs. 4.5% p<0.001, and 3.5% vs.2.9% p<0.001, respectively). Hypochloremia was associated with metabolic alkalosis (3.8% vs.1.4%, p<0.001), while hyperchloremia reflected metabolic acidosis (22% vs.12%, p<0.001). A continuous trend for a positive association from hypochloremia extending to euchloremia and to hyperchloremia was observed for only two conditions: nephrolithiasis and metabolic acidosis.

**Table 1:** Baseline characteristics of study population stratified per serum chloride levels: Hypochloremia (< = 97 mmol/L), Euchloremia (98–106 mmol/L), Hyperchloremia (> = 107 mmol/L) (n = 664253 serum chloride tests of 105655 patients, adjusted to same patients clustering).

Variable		Hypochloremia (n = 25401 tests)	Euchloremia (n = 528412 tests)	Hyperchloremia (n = 110440 tests)	P value Hypochloremia vs. Euchloremia	P value Hyperchloremia vs. Euchloremia
Age, years (mean±SD)		65.5±16	59.4±16	60.1±17	<0.001	<0.001
Gender, female N (%)		13926 (54.8)	295065 (55.9)	67975 (61.6)	0.083	<0.001
**Background diseases**						
Hypertension N (%)		4350 (17.1)	89378 (16.9)	18150 (16.4)	<0.001	0.066
Cardiovascular disease N (%)		3278 (12.9)	38779 (7.3)	8679 (7.9)	0.002	0.001
Diabetes mellitus N (%)		5244 (20.6)	90702 (17.2)	15366 (13.9)	0.064	<0.001
Dyslipidemia N (%)		6510 (25.6)	161353 (30.5)	32236 (29.2)	0.001	0.052
Congestive heart disease N (%)		2060 (8.1)	14722 (2.8)	3333 (3.0)	<0.001	0.018
Chronic kidney disease N (%)		2094 (8.2)	23896 (4.5)	9530 (8.6)	<0.001	<0.001
Malignancy N (%)		1213 (4.8)	25613 (4.8)	6145 (5.6)	0.346	<0.001
Cirrhosis N (%)		105 (0.4)	1486 (0.4)	567 (0.5)	0.348	<0.001
Anemia N (%)		1126 (4.4)	15859 (3.0)	4404 (4.0)	0.005	<0.001
Osteoporosis N (%)		1471 (5.8)	27172 (5.1)	6531 (5.9)	0.954	<0.001
Nephrolithiasis N (%)		536 (2.1)	15294 (2.9)	3844 (3.5)	<0.001	<0.001
**Chronic medications**						
RAS inhibitors N (%)		15973 (62.9)	226705 (42.9)	45408 (41.1)	0.012	<0.001
Diuretics N (%)		11553 (45.5)	103428 (19.6)	20661 (18.7)	<0.001	0.034
BB N (%)		12335 (48.6)	161899 (30.6)	35153 (31.8)	<0.001	0.209
CBB N (%)		10502 (41.3)	131094 (24.8)	29795 (27.0)	<0.001	0.002
**Labs**						
Hyponatremia (<135 mmol/L, n = 557301 tests) N (%)		8571 (49.5) (n = 17301 tests)	9550 (2.1) (n = 449269 tests)	203 (0.2) (n = 90731 tests)	<0.001	<0.001
CKD stages according to MDRD, (mL/min/1.73 m^2^) N (%)	≥ 90	9286 (36.6)	226279 (42.9)	36873 (33.4)	0.043	<0.001
	60–90	9222 (36.4)	222829 (42.2)	44310 (40.2)	0.202	<0.001
	30–60	4314 (17.0)	61899 (11.7)	21009 (19.0)	0.003	<0.001
	<30	2544 (10.0)	16783 (3.2)	8138 (7.4)	<0.001	<0.001
Hypoalbuminemia (<3.5 gr/dL) N (%)		3758 (14.8)	31911 (6.0)	9882 (8.9)	0.001	<0.001
Metabolic acidosis (PH<7.38 and HCO3<23), N (%) (N = 240901 tests))		1668 (11.1) (n = 15028)	21921 (12.0) (n = 182832)	9484 (22.0) (n = 43049)	0.069	<0.001
Metabolic alkalosis (PH>7.42 and HCO3>30), N (%)(N = 240901 tests)		569 (3.8) (n = 15028)	2582 (1.4) (n = 182832)	357 (0.8) (n = 43049)	<0.001	<0.001

Medication use during periods of hypochloremia was characterized by significantly higher proportions of RAS inhibitors, diuretics, beta-blockers and calcium channel blockers (62.9% vs. 42.9 p = 0.012, 45.5% vs.19.6% p<0.001, 48.6% vs.30.6% p<0.001 and 41.3% vs.24.8% p<0.001, respectively).

We further divided the hypochloremic group, into hypochloremia/hyponatremia and hypochloremia/eunatremia groups (hypochloremia/hypernatremia combination is practically non-existent (database is available online)) (Table 2).

**Table 2:** Sub-population of hypochloremia (< = 97 mmol/L), per natremia status (n = 17301 tests, adjusted to same patients clustering).

Variable		Hyponatremia (n = 8571 tests)	Eunatremia (n = 8730 tests)	P value
Age, years (mean± SD)		70.0±14.7	67.4±15	<0.001
Gender, female N (%)		4865 (56.8)	4697 (53.9)	0.047
Hypertension N (%)		2282 (26.6)	1959 (22.4)	0.534
Cardiovascular disease N (%)		1626 (19.0)	1528 (17.5)	0.551
Diabetes mellitus N (%)		2585 (30.2)	2497 (28.6)	0.745
Dyslipidemia N (%)		3395 (39.6)	2953 (33.8)	<0.001
Congestive heart disease N (%)		956 (11.2)	1009 (11.6)	0.130
Chronic kidney disease N (%)		967 (11.3)	1045 (12.0)	0.734
Malignancy N (%)		710 (8.3)	459 (5.3)	<0.001
Cirrhosis N (%)		70 (0.8)	32 (0.4)	0.003
Anemia N (%)		592 (6.9)	486 (5.6)	0.135
Osteoporosis N (%)		805 (9.4)	640 (7.3)	0.002
RAS inhibitors N (%)		5598 (65.3)	5686 (65.1)	0.406
Diuretics N (%)		3787 (44.2)	4736 (54.2)	<0.001
BB N (%)		4379 (51.1)	4535 (51.9)	0.225
CBB N (%)		3633 (42.4)	3745 (42.9)	0.529
CKD stages according to MDRD, (mL/min/1.73 m^2^) N (%)	≥ 90	3352 (39.2)	3076 (35.3)	0.004
	60–90	2567 (30.0)	2871 (32.9)	0.488
	30–60	1505 (17.6)	1672 (19.2)	0.133
	<30	1126 (13.2)	1098 (12.6)	0.635
Hypoalbuminemia (<3.5 gr/dL) N (%)		1907 (22.2)	1230 (14.1)	<0.001
Metabolic acidosis (PH<7.38 and HCO3<23), N (%) (N = 10960 tests)		731 (13.1)	451 (8.5)	<0.001
Metabolic alkalosis (PH>7.42 and HCO3>30), N (%) (N = 10960 tests)		172 (3.1)	279 (5.3)	<0.001

This stratification of hypochloremia demonstrated that within the group of hypochloremia, it is chloride depletion that accounts for metabolic alkalosis (5.3% vs. 3.1%, p = 0.009). As expected, in patients taking diuretics chloride depletion was more common (54.2% vs. 44.2% p<0.001). In contrast, in patients with malignancy chloride depletion was less common (8.3% vs.5.3% p<0.001.

### Outcome

**Fig 2:**
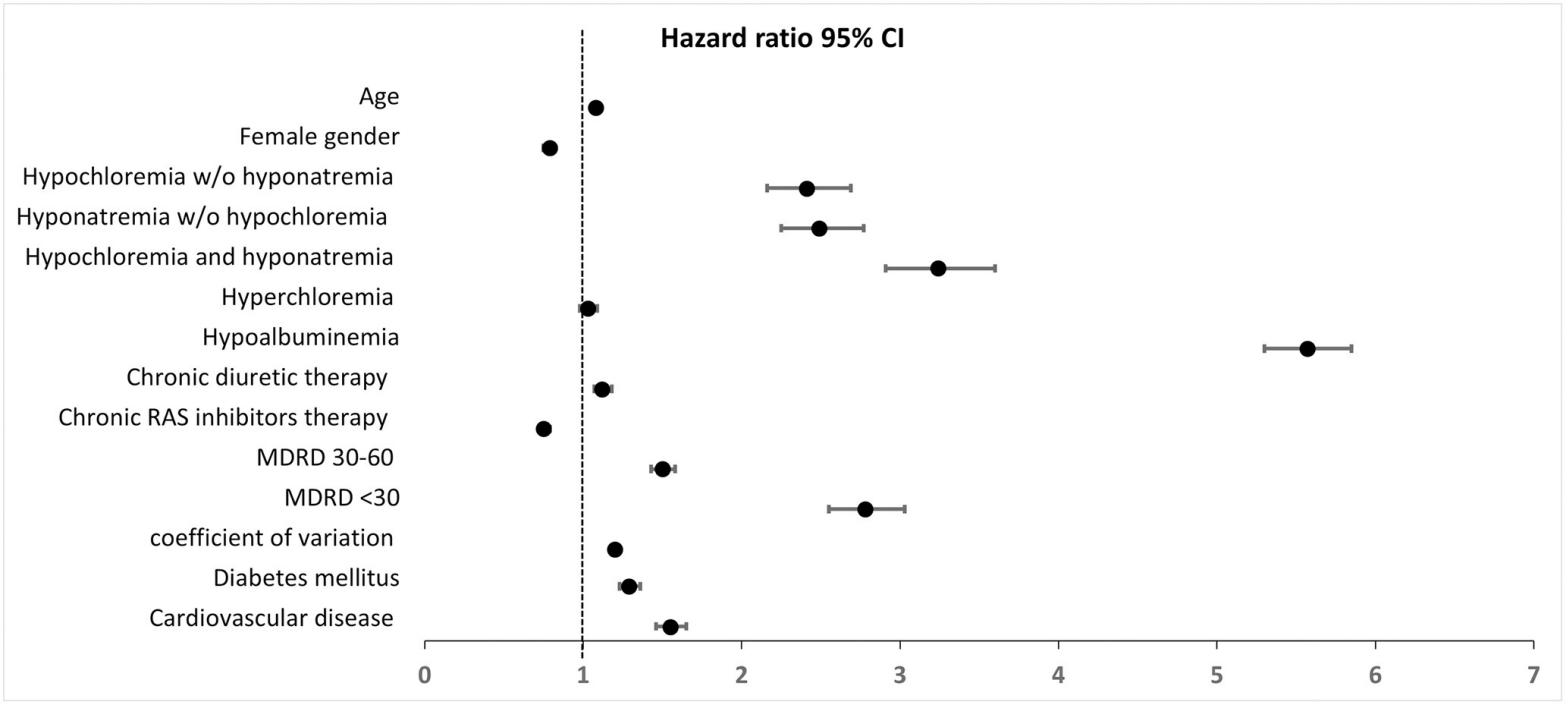


During a median follow up duration of 10.5 years, 11,694 subjects out of 105,655 died (Kaplan Meier rate of 11%). In Cox proportional hazards multivariate analysis, hypochloremia (≤97 mmol/L) was an independent strong predictive factor for all-cause mortality. Both depletion hypochloremia (HR = 2.41, 95% CI 2.16–2.69) and hyponatremic hypochloremia (HR = 3.24, 95%CI 2.91–3.60), were risk factors after adjustment to age, sex, co-morbidities, eGFR (MDRD), medication use (diuretics and angiotensin-aldosterone system inhibitors), hypoalbuminemia and coefficient of chloride variation (Table 3, Fig 2). Crude hyperchloremia (≥107 mmol/L) did not appear to confer a risk for all-cause mortality (HR = 1.03 95% CI 0.98–1.09, p = 0.231).

**Table 3:** Multivariate analysis (Cox regression with same patients clustering) for all-cause mortality (n = 556,595 tests of 105,207 patients).

Variable		HR	95% CI	P value
Age (at first chloride test)		1.08	1.07–1.08	<0.001
Female gender		0.79	0.75–0.82	<0.001
Hypochloremia (< = 97 mmol/L) -hyponatremia (<135 mmol/L) groups (with no hypochloremia no hyponatremia as reference)	Hypochloremia w/o hyponatremia	2.41	2.16–2.69	<0.001
	Hyponatremia w/o hypochloremia	2.49	2.25–2.77	<0.001
	Hypochloremia + hyponatremia	3.24	2.91–3.60	<0.001
Hyperchloremia (≥107 mmol/L)		1.03	0.98–1.09	0.231
Hypoalbuminemia (<3.5 g/dl)		5.57	5.30–5.85	<0.001
Chronic diuretic therapy		1.12	1.07–1.18	<0.001
Chronic RAS inhibitors therapy		0.75	0.72–0.79	<0.001
MDRD (with MDRD>60 as reference group)	30–60	1.50	1.43–1.58	<0.001
	<30	2.78	2.55–3.03	<0.001
Coefficient of variation (CV) (quartiles)		1.20	1.18–1.23	<0.001
Diabetes mellitus		1.29	1.23–1.36	<0.001
Cardiovascular disease		1.55	1.46–1.65	<0.001

Patient-level serum chloride level variations were associated with elevated mortality risk (HR for coefficient of variation quartile 1.20 95% CI 1.18–1.23 p<0.001) (Table 3, Fig 2). Of note, while diuretic use was associated with a slightly higher mortality risk, the use of RAS inhibitors was associated with better survival (HR 0.75 CI 0.72–0.79).

### Serum chloride optimal range

To define the low and high chloride cutoffs associated with excess mortality risk, we performed a sensitivity analysis (Tables 4 and 5).

**Table 4:** Multivariate analysis (Cox regression with same patients clustering) for all-cause mortality risk of hypochloremia cutoffs (n = 556595 tests of 105207 patients).

Variable	HR	95% CI	P value
Hypochloremia (≤ 95 mmol/L) (n = 7449(	2.71	2.17–3.37	<0.001
Hypochloremia (≤ 96 mmol/L) (n = 11239)	2.64	2.26–3.07	<0.001
**Hypochloremia (≤ 97** mmol/L**) (n =** 17301)	2.41	2.16–2.70	<0.001
Chloride (≤ 98 mmol/L) (n = 26827)	2.18	2.01–2.37	<0.001
Chloride (≤ 99 mmol/L) (n = 42218)	1.95	1.83–2.09	<0.001
Chloride (≤ 100 mmol/L) (n = 66986)	1.78	1.68–1.88	<0.001
Chloride (≤ 101 mmol/L) (n = 106293)	1.63	1.55–1.71	<0.001
Chloride (≤ 102 mmol/L) (n = 163257)	1.50	1.43–1.58	<0.001
Chloride (≤ 103 mmol/L) (n = 238160)	1.41	1.34–1.48	<0.001
Chloride (≤ 104 mmol/L) (n = 322672)	1.30	1.23–1.37	<0.001
Chloride (≤ 105 mmol/L) (n = 402751)	1.22	1.13–1.31	<0.001
Chloride (≤ 106 mmol/L) (n = 465977)	0.38	0.35–0.41	<0.001

*adjusted to model variables (Table 3)

**Table 5:** Multivariate analysis (Cox regression with same patients clustering) for all-cause mortality (n = 556595 tests of 105207 patients) of hyperchloremia cutoffs.

Variable	HR	95% CI	P value
Chloride (≥105 mmol/L) (n = 233930)	0.88	0.84–0.92	<0.001
Chloride (≥106 mmol/L) (n = 153853)	0.96	0.92–1.01	0.083
Chloride **(≥107** mmol/L**) (**n **=** 90731**)**	1.03	0.98–1.09	0.231
Hyperchloremia (≥108 mmol/L) (n = 48978)	1.14	1.06–1.21	<0.001
Hyperchloremia (≥109 mmol/L) (n = 25094)	1.21	1.11–1.31	<0.001

*adjusted to model variables (Table 3)

**Fig 3:**
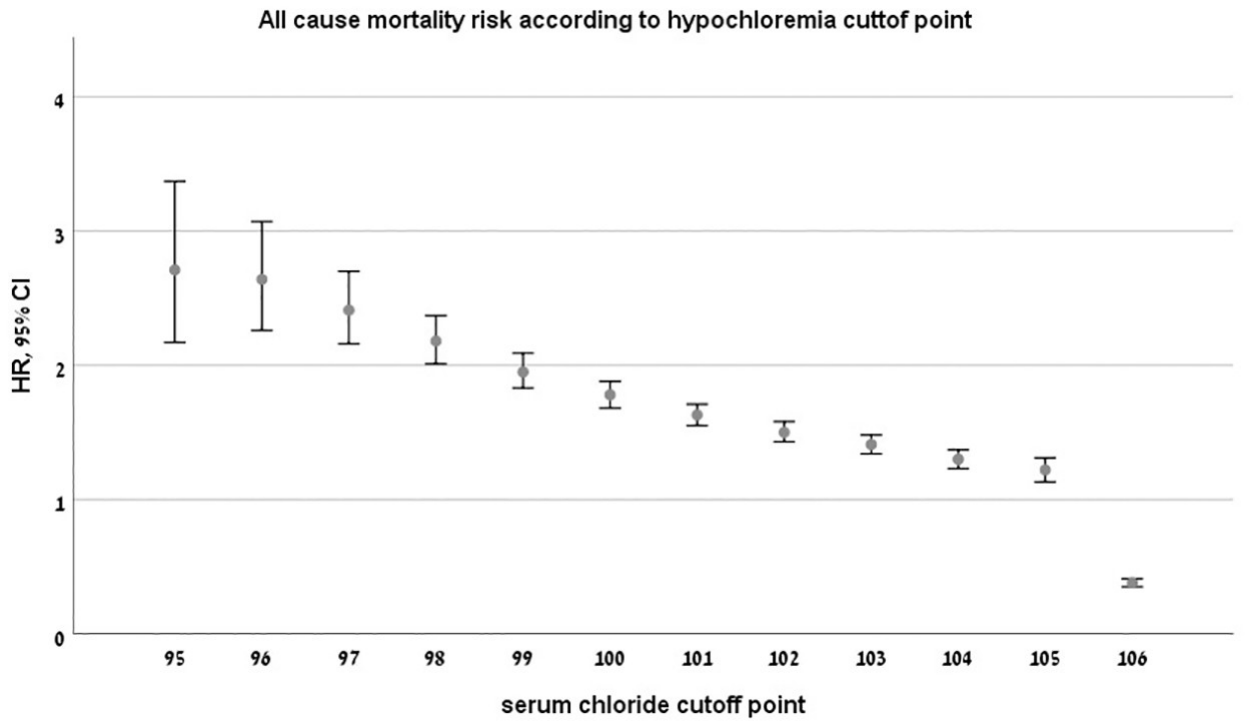


For hypochloremia, mortality risk decreased for every 1 mmol/L rise in serum chloride level well within the "normal" range values (HR 2.71 95% CI 2.17–3.37 for cutoff of ≤95 mmol/l, HR 2.41 95% 2.16–2.70 for cutoff of ≤97 mmol/l (normal range cutoff), up to chloride ≤ 105 mmol/L (HR 1.22 95% CI 1.13–1.31 p<0.001, Table 4, Fig 3). The risk declined abruptly at a serum chloride level of 106 mmol/l (HR 0.38 95% CI 0.35–0.41 p<0.001). For hyperchloremia, a cutoff level of ≥108 mmol/L was associated with elevated mortality risk (Table 5, HR 1.14 95% CI 1.03–1.21 p<0.001)). Thus, crude serum chloride values at 106–107 mmol/l appear to be associated with the lowest mortality risk in the general population.

### Serum chloride and acid-base status

**Fig 4:**
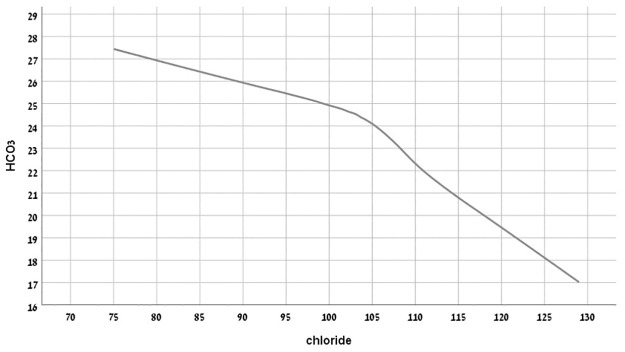


We next assessed the relationship between chloride levels and acid-base status in non-hospitalized patients with the closest -available venous blood bicarbonate level (only test in this study taken at hospital, 240,909 tests of 29,581 patients). HCO_3_ and serum chloride correlation (Fig 4) indicated that metabolic alkalosis correlates with chloride values (adjusted to serum sodium) ≤ 90 mmol/L and that metabolic acidosis correlates with chloride values (adjusted to serum sodium) ≥ 110 mmol/L. Sensitivity analysis confirmed similar prognostic value for hypochloremia in the subpopulation where bicarbonate levels were available (Table 4s supplement in S1 File).

## Discussion

In the current population-based retrospective cohort study, the largest chloride study so far including more than one hundred thousand patients during a median follow up time of 10.8 years, we found that hypochloremia in the general population is associated with an elevated all-cause mortality risk, after adjusting for age, sex, co-morbidities, diuretics and RAS inhibitors therapy, serum sodium, albumin levels and estimated GFR.

Serum chloride was traditionally viewed as a passive anion linked to sodium levels. However, in 1998 the first study evaluating hypochloremia found an association with mortality in the general population [1]. Following studies in CHF patients corroborated the strong predictive value of serum chloride [2]. Next, in a cohort of hypertensives [7] hypochloremia, but not hyperchloremia, also predicted mortality. In contrast, in a cohort of CKD patients both hypochloremia and hyperchloremia were associated with mortality [9]. Because hyponatremia is a well-known prognostic factor for mortality, adjustment for serum osmolarity, and its proxy the serum sodium level, is required. To this end, the study population was divided into 4 groups, based on the hyponatremia status. Our results point out that only half of hypochloremia periods were hyponatremic hypochloremia. In a previous study, among all patients with hypochloremia, 48.7% had hyponatremia [6], compared to 49.5% in the current study. In multivariate analysis, we found that both hyponatremic hypochloremia (low chloride, low sodium serum levels) and chloride depletion (low chloride, normal sodium serum levels) were independently associated with increased mortality risk (Table 3, Fig 2). Thus, both water excess and chloride depletion (indicating either volume depletion or metabolic alkalosis, or both) are major risk markers of mortality.

The mechanism of the risk reflected by hypochloremia is unknown and may be multi-factorial. Chloride may play a major role in extracellular volume regulation [23–26]. Because the risk associated with low serum chloride was independent of serum sodium, pH and bicarbonate, low chloride concentration in the glomerular filtrate may induce activation of renin, loop of Henle’s NKCC and distal tubular Na-Cl symporter.

Hyperchloremia, (≥107 mmol/L), was not a statistically significant risk factor. This finding corroborates the finding in a cohort of hypertensives [7] and CHF patients [5] who reported that hyperchloremia ≥107 mmol/l or ≥ 104 respectively, did not predict mortality. Only when hyperchloremia was associated with hyponatremia, possibly indicating extreme hyperchloremia, we found an additional risk (HR for interaction 1.71, p = 0.032), Extreme hyperchloremia is an indicator of metabolic acidosis, as shown in Fig 4. Of note, hyperchloremia of Cl≥108 mmol/l, was independently associated with risk (Tables 4 and 5), thereby compatible with a J-shaped risk for serum chloride in the general population. A J-shaped curve association between mortality and chloride levels has been previously reported in a Japanese CKD population and among USA discharged patients where both hypochloremia and hyperchloremia predicted mortality [9, 10]. In our study, CKD was also associated with both hypo- and hyperchloremia (Table 1). In the context of CKD, serum chloride levels were previously found to better reflect renal function than serum sodium [6].

The normal range for serum chloride is debatable. A previous study suggested that the lower chloride normal range level should be 100 mmol/L [4]. In contrast, our study indicates that even values within the higher spectrum of the normal range (≤ 105 mmol/L) predicted a higher risk of mortality (Fig 2, Table 4). Because lower serum chloride levels appear to be a proxy for either chloride depletion or water excess, even moderate degrees of these two conditions may confer a risk for mortality, as evident by higher mortality associated with chloride levels even within the lower normal range. In contrast, when shifting up the high normal range value to define hyperchloremia above ≥108 or 109 mmol/l, the mortality risk became statistically significant (HR 1.14 P<0.001 and 1.21 p<0.001, respectively, Table 5), possibly reflecting metabolic acidosis. Thus, in our general population the optimal lower chloride serum level appears to be higher than recommended, i.e. 106–107 mmol/l, rather than 96–98 mmol/l.

This study had several limitations. First, the observational retrospective nature of this study may indicate association rather than causality. Second, because our basic assumption was that the frequency of chloride tests was higher among patients with abnormal chloride levels, we designed the study to calculate the periods with high/ low/ normal levels according to the last test value until the next test was taken, which may skew the results. Third, the correlation between chloride levels and blood bicarbonate may be hampered by time difference between collection of community-based and hospital based blood chemistry and gases, respectively. Finally, because urine electrolytes were not available the group of hyponatremic hypochloremia remains heterogenous comprising hypovolemia and water excess in unknown ratios.

The strengths of the study include i. its size, the largest so far. ii. Its design focusing on serum chloride in the general population. iii. collection of longitudinal measurements of at least 3 serum chloride tests differing from some previous studies based on a single measured serum chloride test. iv. a long median follows up of 10.5 years, enabling the separate evaluation of the risk related to distinct periods of serum chloride abnormalities.

In conclusion, in this population-based study outpatient hypochloremia was associated with excess all-cause mortality, either when reflecting chloride depletion or water dilution. Even within the normal range the risk of lower chloride level was substantial. Thus, because serum chloride tests are part of the routine clinical evaluation, attention to serum chloride levels independently of sodium levels may identify subjects at risk.

## Supporting information

S1 Data (XLSB)

S1 File (DOCX)

## Abbreviations

AG: anion gap
BB: beta blockers
CCB: calcium channel blockers
CHF: congestive heart disease
CKD: chronic kidney disease
ED: emergency department
ESRD: end stage renal disease
FU: follow up
GEE: generalized estimating equation
GFR: glomerular filtration rate
HMO: health maintenance organization
HR: hazard ratio
ICU: intensive care unit
IQR: interquartile range
LOWESS: Locally Weighted Scatterplot Smoothing
MDRD: Modification of Diet in Renal Disease
MM: multiple myeloma
Mmol: milli mo
RAS: renin-angiotensin- aldosterone system
SD: standard deviation
SUMC: Soroka University Medical Center
TPN: Total parenteral nutrition
