# Supplementary material for: Community-based serum chloride abnormalities predict mortality risk
Source: PLoS One. 2023 Feb 21;18(2):e0279837. doi: 10.1371/journal.pone.0279837 (PMC9942956; doi:10.1371/journal.pone.0279837)
Supplement: S1 File — (DOCX) [file pone.0279837.s002.docx]

Supplementary Tables

Table 1s-a: Multivariate analysis (Cox regression with same patients clustering) for all-cause mortality among patients with chronic cardiovascular disease (n=50166 tests of 9140 patients):

| Variable | | | HR | 95% CI | P value |
| --- | --- | --- | --- | --- | --- |
| Age (at first chloride test) | | | 1.06 | 1.05-1.07 | <0.001 |
| Female gender | | | 0.92 | 0.84-1.01 | 0.086 |
| Hypochloremia (<=97 mmol/L) -hyponatremia (<135 mmol/L) groups (with no hypochloremia no hyponatremia as reference) | | Hypochloremia w/o hyponatremia | 1.98 | 1.54-2.55 | <0.001 |
|  |  | hyponatremia w/o hypochloremia | 2.11 | 1.72-2.59 | <0.001 |
|  |  | Hypochloremia + hyponatremia | 2.38 | 1.96-2.90 | <0.001 |
| Hyperchloremia (≥107 mmol/L) | | | 0.98 | 0.88-1.11 | 0.835 |
| Hypoalbuminemia (<3.5 g/dl) | | | 3.74 | 3.37-4.15 | <0.001 |
| Chronic diuretic therapy | | | 1.48 | 1.34-1.64 | <0.001 |
| Chronic RAS inhibitors therapy | | | 0.78 | 0.70-0.87 | <0.001 |
| MDRD (with MDRD>60 as reference group) | 30-60 | | 1.39 | 1.25-1.54 | <0.001 |
|  | <30 | | 2.45 | 2.11-2.83 | <0.001 |
| Coefficient of variation (CV) (quartiles) | | | 1.18 | 1.12-1.24 | <0.001 |
| Diabetes mellitus | | | 1.14 | 1.04-1.25 | 0.004 |

Table 1s-b: Multivariate analysis (Cox regression with same patients clustering) for all-cause mortality among patients with **no** chronic cardiovascular disease (n=506429 tests of 102000 patients):

| Variable | | | HR | 95% CI | P value |
| --- | --- | --- | --- | --- | --- |
| Age (at first chloride test) | | | 1.08 | 1.07-1.08 | <0.001 |
| Female gender | | | 0.77 | 0.74-0.81 | <0.001 |
| Hypochloremia (<=97 mmol/L) -hyponatremia (<135 mmol/L) groups (with no hypochloremia no hyponatremia as reference) | | Hypochloremia w/o hyponatremia | 2.57 | 2.30-2.88 | <0.001 |
|  |  | hyponatremia w/o hypochloremia | 2.57 | 2.27-2.90 | <0.001 |
|  |  | Hypochloremia + hyponatremia | 3.51 | 3.09-3.99 | <0.001 |
| Hyperchloremia (≥107 mmol/L) | | | 1.03 | 0.97-1.10 | 0.275 |
| Hypoalbuminemia (<3.5 g/dl) | | | 6.12 | 5.79-6.46 | <0.001 |
| Chronic diuretic therapy | | | 1.07 | 1.01-1.12 | 0.023 |
| Chronic RAS inhibitors therapy | | | 0.75 | 0.71-0.78 | <0.001 |
| MDRD (with MDRD>60 as reference group) | 30-60 | | 1.52 | 1.44-1.61 | <0.001 |
|  | <30 | | 2.89 | 2.62-3.19 | <0.001 |
| Coefficient of variation (CV) (quartiles) | | | 1.21 | 1.18-1.23 | <0.001 |
| Diabetes mellitus | | | 1.31 | 1.23-1.39 | <0.001 |

Table 2s-a: Multivariate analysis (Cox regression with same patients clustering) for all-cause mortality among patients with diabetes mellitus (n=110388 tests of 22295 patients):

| Variable | | | HR | 95% CI | P value |
| --- | --- | --- | --- | --- | --- |
| Age (at first chloride test) | | | 1.07 | 1.06-1.07 | <0.001 |
| Female gender | | | 0.86 | 0.79-0.93 | <0.001 |
| Hypochloremia (<=97 mmol/L) -hyponatremia (<135 mmol/L) groups (with no hypochloremia no hyponatremia as reference) | | Hypochloremia w/o hyponatremia | 2.34 | 1.97-2.80 | <0.001 |
|  |  | hyponatremia w/o hypochloremia | 2.05 | 1.69-2.49 | <0.001 |
|  |  | Hypochloremia + hyponatremia | 2.84 | 2.36-3.42 | <0.001 |
| Hyperchloremia (≥107 mmol/L) | | | 1.02 | 0.91-1.13 | 0.719 |
| Hypoalbuminemia (<3.5 g/dl) | | | 5.01 | 4.56-5.50 | <0.001 |
| Chronic diuretic therapy | | | 1.26 | 1.15-1.38 | <0.001 |
| Chronic RAS inhibitors therapy | | | 0.74 | 0.67-0.81 | <0.001 |
| MDRD (with MDRD>60 as reference group) | 30-60 | | 1.49 | 1.35-1.63 | <0.001 |
|  | <30 | | 2.40 | 2.09-2.75 | <0.001 |
| Coefficient of variation (CV) (quartiles) | | | 1.20 | 1.16-1.38 | <0.001 |
| Cardiovascular disease | | | 1.52 | 1.38-1.66 | <0.001 |

Table 2s-b: Multivariate analysis (Cox regression with same patients clustering) for all-cause mortality among patients with **no** diabetes mellitus (n=446207 tests of 97156 patients):

| Variable | | | HR | 95% CI | P value |
| --- | --- | --- | --- | --- | --- |
| Age (at first chloride test) | | | 1.08 | 1.07-1.08 | <0.001 |
| Female gender | | | 0.77 | 0.73-0.80 | <0.001 |
| Hypochloremia (<=97 mmol/L) -hyponatremia (<135 mmol/L) groups (with no hypochloremia no hyponatremia as reference) | | Hypochloremia w/o hyponatremia | 2.44 | 2.12-2.80 | <0.001 |
|  |  | hyponatremia w/o hypochloremia | 2.72 | 2.41-3.08 | <0.001 |
|  |  | Hypochloremia + hyponatremia | 3.42 | 2.99-3.88 | <0.001 |
| Hyperchloremia (≥107 mmol/L) | | | 1.04 | 0.97-1.10 | 0.248 |
| Hypoalbuminemia (<3.5 g/dl) | | | 5.74 | 5.42-6.09 | <0.001 |
| Chronic diuretic therapy | | | 1.09 | 1.03-1.15 | 0.003 |
| Chronic RAS inhibitors therapy | | | 0.76 | 0.72-0.80 | <0.001 |
| MDRD (with MDRD>60 as reference group) | 30-60 | | 1.51 | 1.42-1.59 | <0.001 |
|  | <30 | | 2.97 | 2.67-3.31 | <0.001 |
| Coefficient of variation (CV) (quartiles) | | | 1.21 | 1.18-1.23 | <0.001 |
| Cardiovascular disease | | | 1.57 | 1.45-1.70 | <0.001 |

| Variable | | | HR | 95% CI | P value |
| --- | --- | --- | --- | --- | --- |
| Age (at first chloride test) | | | 1.08 | 1.08-1.09 | <0.001 |
| Female gender | | | 0.85 | 0.80-0.89 | <0.001 |
| Hypochloremia (<=97 mmol/L) -hyponatremia (<135 mmol/L) groups (with no hypochloremia no hyponatremia as reference) | | Hypochloremia w/o hyponatremia | 2.20 | 1.91-2.54 | <0.001 |
|  |  | hyponatremia w/o hypochloremia | 2.28 | 2.00-2.60 | <0.001 |
|  |  | Hypochloremia + hyponatremia | 2.76 | 2.43-3.13 | <0.001 |
| Hyperchloremia (≥107 mmol/L) | | | 1.06 | 0.98-1.38 | 0.125 |
| Hypoalbuminemia (<3.5 g/dl) | | | 5.03 | 4.71-5.36 | <0.001 |
| Chronic diuretic therapy | | | 1.16 | 1.10-1.22 | <0.001 |
| MDRD (with MDRD>60 as reference group) | 30-60 | | 1.40 | 1.32-1.49 | <0.001 |
|  | <30 | | 2.73 | 2.46-3.02 | <0.001 |
| Coefficient of variation (CV) (quartiles) | | | 1.16 | 1.13-1.19 | <0.001 |
| Diabetes mellitus | | | 1.27 | 1.20-1.35 | <0.001 |
| Cardiovascular disease | | | 1.61 | 1.51-1.72 | <0.001 |

Table 3s-a: Multivariate analysis (Cox regression with same patients clustering) for all-cause mortality among patients with chronic RAS inhibitors treatment (n=240645 tests of 37717 patients):

| Variable | | | HR | 95% CI | P value |
| --- | --- | --- | --- | --- | --- |
| Age (at first chloride test) | | | 1.07 | 1.07-1.08 | <0.001 |
| Female gender | | | 0.72 | 0.67-0.76 | <0.001 |
| Hypochloremia (<=97 mmol/L) -hyponatremia (<135 mmol/L) groups (with no hypochloremia no hyponatremia as reference) | | Hypochloremia w/o hyponatremia | 2.77 | 2.33-3.30 | <0.001 |
|  |  | hyponatremia w/o hypochloremia | 2.78 | 2.33-3.31 | <0.001 |
|  |  | Hypochloremia + hyponatremia | 4.08 | 3.35-4.95 | <0.001 |
| Hyperchloremia (≥107 mmol/L) | | | 1.01 | 0.93-1.10 | 0.835 |
| Hypoalbuminemia (<3.5 g/dl) | | | 6.26 | 5.80-6.76 | <0.001 |
| Chronic diuretic therapy | | | 1.07 | 0.97-1.18 | 0.160 |
| MDRD (with MDRD>60 as reference group) | 30-60 | | 1.69 | 1.54-1.85 | <0.001 |
|  | <30 | | 2.80 | 2.40-3.28 | <0.001 |
| Coefficient of variation (CV) (quartiles) | | | 1.25 | 1.21-1.29 | <0.001 |
| Diabetes mellitus | | | 1.35 | 1.23-1.29 | <0.001 |
| Cardiovascular disease | | | 1.44 | 1.27-1.64 | <0.001 |

Table 3s-b: Multivariate analysis (Cox regression with same patients clustering) for all-cause mortality among patients with **no** chronic RAS inhibitors treatment (n=315950 tests of 67490 patients

| Variable | | | HR | 95% CI | P value |
| --- | --- | --- | --- | --- | --- |
| Age (at first chloride test) | | | 1.06 | 1.06-1.06 | <0.001 |
| Female gender | | | 0.83 | 0.79-0.87 | <0.001 |
| Hypochloremia (<=97 mmol/L) -hyponatremia (<135 mmol/L) groups (with no hypochloremia no hyponatremia as reference) | | Hypochloremia w/o hyponatremia | 2.33 | 2.08-2.60 | <0.001 |
|  |  | hyponatremia w/o hypochloremia | 2.31 | 2.06-2.59 | <0.001 |
|  |  | Hypochloremia + hyponatremia | 2.81 | 2.50-3.17 | <0.001 |
| Hyperchloremia (≥107 mmol/L) | | | 0.96 | 0.90-1.02 | 0.188 |
| Hypoalbuminemia (<3.5 g/dl) | | | 4.67 | 4.43-4.93 | <0.001 |
| Metabolic acidosis (PH<7.38 and HCO3<23) | | | 1.45 | 1.36-1.54 | <0.001 |
| Metabolic alkalosis (PH>7.42 and HCO3>30) | | | 1.29 | 1.08-1.53 | 0.005 |
| Chronic diuretic therapy | | | 1.04 | 0.99-1.10 | 0.110 |
| Chronic Ras inhibitors therapy | | | 0.66 | 0.63-0.70 | <0.001 |
| MDRD (with MDRD>60 as reference group) | 30-60 | | 1.33 | 1.26-1.40 | <0.001 |
|  | <30 | | 2.17 | 1.99-2.36 | <0.001 |
| Coefficient of variation (CV) (quartiles) | | | 1.08 | 1.06-1.11 | <0.001 |
| Diabetes mellitus | | | 1.29 | 1.22-1.37 | <0.001 |
| Cardiovascular disease | | | 1.50 | 1.41-1.60 | <0.001 |

Table 4s: Multivariate analysis (Cox regression with same patients clustering) for all-cause mortality among patients with available acid-base status (n=240901 tests of 29580 p
